# Identification of an ADME-related gene for forecasting the prognosis and responding to immunotherapy in sarcomas

**DOI:** 10.1186/s40001-023-01624-3

**Published:** 2024-01-11

**Authors:** Jianlong Wang, Guowei Wang, Tianrui Hu, Hongyi Wang, Yong Zhou

**Affiliations:** 1 Department of Orthopedics, Third Xiangya Hospital, Central South University, Changsha, 410013 Hunan China; 2 Medical College, Hunan Normal University, Changsha, 410013 Hunan China

**Keywords:** Sarcomas, Osteosarcoma, ADME, PPARD, Immunotherapy

## Abstract

There are more than 170 subtypes of sarcomas (SARC), which pose a challenge for diagnosis and patient management. Relatively simple or complex karyotypes play an indispensable role in the early diagnosis and effective treatment of SARC. The genes related to absorption, distribution, metabolism, and excretion (ADME) of a drug can serve as prognostic biomarkers of cancer and potential drug targets. In this study, a risk score signature was created. The SARC cohort was downloaded from The Cancer Genome Atlas (TCGA) database, and divided into high-risk group and low-risk group according to the median value of risk score. Compared with high-risk group, low-risk group has a longer survival time, which is also verified in osteosarcoma cohort from Therapeutically Applicable Research to Generate Effective Treatments (TARGET) database. In addition, the relationship between the signature and immunophenotypes, including status of immune cell infiltration and immune checkpoint expression, was explored. Then, we found that high-risk group is in immunosuppressive status. Finally, we verified that PPARD played a role as a carcinogen in osteosarcoma, which provided a direction for targeted treatment of osteosarcoma in the future. Generally speaking, the signature can not only help clinicians predict the prognosis of patients with SARC, but also provide a theoretical basis for developing more effective targeted drugs in the future.

**Supplementary Information:**

The online version contains supplementary material available at 10.1186/s40001-023-01624-3.

## Introduction

Sarcomas (SARC) are a heterogeneous group of tumors that arise from mesenchymal tissues such as bone, cartilage, muscle and other connective tissues [1]. Nowadays, two broad genetic groups of SARC, including simple karyotypes and highly complex karyotypes, have been identified [2]. In SARC types with complex cytogenetic changes, the molecular alterations are diverse. According to the latest WHO classification, there are more than 170 subtypes of SARC that vary in pathology, clinical presentation, molecular features and response to treatment [3, 4]. Based on histopathological criteria and the type of tissue in which they predominantly present, 80% of sarcomas are classified as soft tissue sarcomas (STS), 15% as osteosarcomas and 5% as gastrointestinal mesenchymal tumors. In terms of prevalence, they account for less than 1% of adult cancers, but one fifth of solid malignancies in children [5]. However, the prevalence of SARC may be underestimated because SARC that develop in parenchymal organs are usually attributed to the affected organ rather than the surrounding connective or supporting tissue [6]. Misdiagnosis due to their difficulty in differentiation from other malignancies, delayed treatment due to early asymptomatic, and their heterogeneity, aggressiveness, and resistance to current therapeutic options make the challenging of clinical management of SARC [7]. Given the heterogeneity of SARC they are treated with nearly all known treatment modalities including targeted therapy, immunotherapy, radiation and chemotherapy with the combinations related to specific tumor subsets. Notably, one-third of patients develop metastases and approximately 20% of SARC recurrence, and these patients who develop recurrence and metastases respond poorly to radiation and chemotherapy [8].

298 genes related to absorption, distribution, metabolism, and excretion (ADME) were identified by the PharmaADME Consortium [9]. ADME-related genes consist mainly of genes encoding phase I and II drug metabolizing enzymes, drug transport proteins and modifiers [10]. ADME genes are involved in the metabolism, transport and removal of endogenous and exogenous substances such as steroid hormones, bile acids and carcinogens that may promote cancer development [11, 12]. ADME-related genes affect the ability of the liver to metabolize and clear drugs, thereby affecting the efficacy of drug therapy and are associated with anticancer drug resistance [13, 14]. Glutathione S-transferases (GSTs) and ATP Binding Cassette transporter B1 (ABCB1) affect the sensitivity of tumor cells to chemotherapeutic drugs by transporting drug components to the extracellular compartment to reduce their intracellular accumulation [15, 16]. The expression of ABCB6 enhances hepatoma cell proliferation and tumorigenicity by targeting the cell cycle [17]. SOD1 can maintain ROS levels below a threshold to support oncogene dependent proliferation during breast tumor formation [18].

In addition to the fact that ADME-related genes can influence the degree of tumor cell tolerance to chemotherapeutic agents, previous studies have demonstrated the role of ADME-related genes as prognostic cancer biomarkers and therapeutic targets. ABCB1 and ABCC2 promote adriamycin efflux from soft tissue sarcoma cells and reduce drug-induced intracellular accumulation, toxicity and immunogenic cell death [19]. RXRA inhibits rhabdomyosarcoma cell proliferation [20]. Overexpression of Carbonyl reductase 1 (CBR1) inhibits malignant behavior and epithelial mesenchymal transition by suppressing TGF-β signaling in uterine smooth muscle sarcoma cells [21]. Through bioinformatic analysis, Hu et al. determined that the ADME gene predicted overall survival (OS) for a lot of cancer types [22]. Sunitinib, an oral multi-receptor tyrosine kinase inhibitor (TKI), is currently used to treat imatinib-resistant/intolerant gastrointestinal mesenchymal tumors and primarily metabolized by CYP3A4 to N-desethyl sunitinib [23].

In this study, to define an ADME-related signature, transcriptome data and clinical data of SARC, which were downloaded from The Cancer Genome Atlas (TCGA), were used to perform the differentially expressed, univariate Cox (uni-Cox), least absolute shrinkage and selection operator (LASSO) and multivariate Cox (multi-Cox) regression analysis. The signature could predict survival outcomes of SARC and was validated by osteosarcoma data in TARGET database. What is more, we verified the expression of PPARD in osteosarcoma and its function at the cellular level. Finally, immune cell infiltration, immunotherapy response, and clustering analyses were explored between the high-risk and low-risk groups. Our results suggest that ADME-related signature can predict prognosis, immune cell infiltration status and treatment response in SARC.

## Materials and methods

### Data collection

We obtained RNA sequencing and clinical data of SARC from TCGA (https://portal.gdc.cancer.gov/) and data of osteosarcoma (TARGET-OS) from UCSC Xena (https://xena.ucsc.edu/), respectively [24]. The package of "sva" was used for batch calibration [25]. Patients without survival information were excluded. ADME-related genes were obtained from the PharmaADME Consortium (http://www.pharmaadme.org) (Additional file 1: Table S1).

### Extraction of prognosis-related ADME Genes

Kaplan–Meier (K–M) survival analysis and univariate Cox (uni-Cox) regression analysis were performed to screen prognosis-related genes (*p* < 0.05). The prognosis-related ADME genes (PARGs) were confirmed by intersection of ADME-related genes and prognosis-related genes.

### Identification of PARGs signatures

We developed a possible risk score signature for SARC by LASSO and multi-Cox regression analysis [26]. The risk score for each SARC patient was calculated as follows:

$$\mathrm{Risk\,score}={\sum }_{i=1}^{N}(\mathrm{Expi }\times {\text{Coei}})$$

All samples were divided into a train cohort (60%) and a test cohort (40%). Patients were defined as high-risk groups by the median risk score of all patients. K–M survival analysis was performed in the whole cohort, train cohort and test cohort, respectively. In addition, uni-Cox and multi-Cox regression analyses were performed to estimate the prognostic independence of the PARGs signature in SARC.

### Establishment and validation of a nomogram

We use the "rms" package to develop a predictive nomogram. In the nomogram, each variable is matched to a score and the total score is obtained by summing all variables in each sample. The nomogram plot was evaluated using receiver operating characteristic (ROC) curves. Calibration plot of the nomogram plots was used to describe the similarity between the predicted survival events and the virtual observed results.

### Estimation of immune cell infiltration

The immune infiltration status of SARC was analyzed from multiple immune data platforms (http://timer.cistrome.org/). The levels of infiltrating immune cells and immune-related functions were also studied by single-sample gene set enrichment analysis (ssGSEA). Then, to predict the therapeutic effect of immune checkpoint blockers (ICB) in different risk groups, we evaluated the expression of 32 immune checkpoint genes [27, 28] (Additional file 2: Table S2). In addition, we assessed tumor microenvironment (TME) scores (stromal score, immune score and estimated score) by "ESTIMATE" package.

### Immunotherapy and prediction of chemotherapy response

We performed the Tumor Immune Dysfunction and Exclusion (TIDE) algorithm to predict the response to immune checkpoint blockade. The TIDE score for each SARC patient was determined by the online web (https://tide.dfci.harvard.edu/). pRRophetic as an R package, can predict clinical chemotherapy responses by gene expression levels to assess the differences in drug sensitivity between the two risk groups [29].

### Consensus clustering analysis of PARGs

The R package "ConsensusClusterPlus" was used for consensus unsupervised clustering analysis to classify samples into different molecular subtypes based on PARGs expression [30]. K–M survival curve was used to assess the prognosis of the different molecular subtypes. Similarly, TME scores (stromal score, immune score and estimation score) for the different molecular subtypes were also assessed by the ESTIMATE package.

### Cell culture and transfection

MG-63 and Saos-2 cells were purchased from the Biological Cell Unit of Central South University. MG-63 and Saos-2 were cultured at 37 °C in a 5% CO_2_ atmosphere with RPMI 1640 (Gibco, USA) supplemented with 10% FBS (Gibco, USA). The short hairpin RNA (shRNA) of PPARD was designed and synthesized by HANBIO (ShangHai, China). The target sequence of shRNA(sh-#1 and sh-#2) was derived from that reported by previous report [31]. (sh-#1, TCAGGCGGCAGCCTCAACATGGAATGTCG; sh-#2, AGCATCCTCACCGGCAAGTCCAGCCACAA. The shRNA pGFP-V-RS plasmid was provided by HANBIO (ShangHai, China) as a negative control, referred in this study as “nc”. Cultured MG-63 and Saos-2 cells were transfected with shRNA-expressing lentiviruses. The stable knockdown cell lines were selected by 1 μg/ml of puromycin.

### Western blot analysis

Cellular protein was extracted by 1 × RIPA buffer (KeyGEN BioTECH) containing 1% PMSF (KeyGEN BioTECH), and protein samples were separated by 10% SurePAGE gels (KeyGEN Bio TEch, JiangSu,China) and transferred onto a 0.45 μm PVDF membranes (Millipore, CA, USA), blocked with 5% skim milk for 2 h, incubated with primary antibodies at 4 °C overnight and secondary antibodies for 1 h at room temperature, and visualized using an Odyssey CLx Infrared Imaging System (LI-COR Biosciences, NE, USA). Protein abundance was analyzed by ImageJ software (National Institutes of Health, Bethesda, MD, USA). Primary antibody: The rabbit anti-PPARD antibody (1:1000, Cell Signaling Technology, Massachusetts, USA). The mouse anti-GAPDH antibody (1:1000, Prpteintech, Wuhan, China).

### Cellular functional assay

To estimate cell proliferation of MG-63 and Saos-2 cells after PPARD knockdown, logarithmically growing cells were seeded in 96-well plates (2 × 10^3^ cells/well). The Cell Counting Kit-8 (CCK-8) assay was performed according to the manufacturer’s instructions after 12, 24, 48 and 72 h in culture. For the colony-forming assay, MG-63 and Saos-2 transfected with shRNA (0.6 × 10^3^ cells/well) were cultured at 37 °C for 2 weeks and then fixed in 4% paraformaldehyde for overnight and stained overnight with 0.05% crystal violet.

Wound-healing assays are used to assess the metastatic ability after PPARD knockdown in MG-63 and Saos-2 cells.

For wound-healing assays, MG-63 and Saos-2 transfected with shRNA (3 × 10^6^ cells/well) were inoculated in 6-well plates. Using a 1 ml pipette to form a wound in the center of the cell monolayer when the cells reach reaching a confluence of 90%, and then continue to culture with a serum-free medium for 48 h. Microscopy images were acquired at 0 and 48 h, and then the area of gaps was quantified by ImageJ software.

### Statistical analysis

All cellular functional assay, as well as western blot assay, were repeated 3 times independently. Statistical computations were performed using GraphPad Prism 8.0 with student’s t-test. And bioinformatics analysis using the R (version 4.0.4). *p* < 0.05 was considered statistically significant.

## Results

### Constructing PARGs signature in TCGA cohorts

**Fig. 1:**
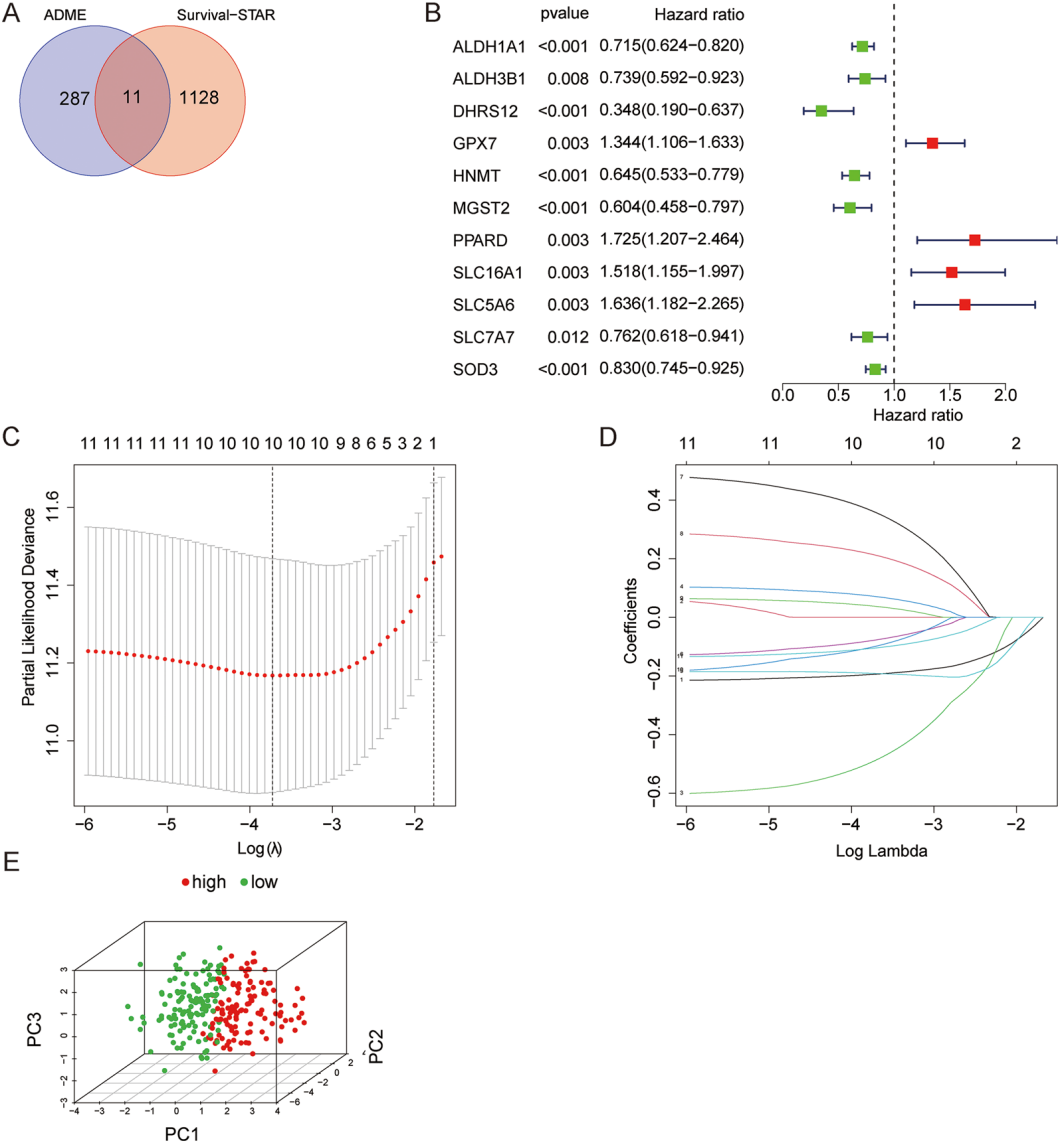
Construction of the ADME-related genes signature. **A** The intersection between ADME-related genes and prognosis-related genes. **B** The univariate Cox regression analysis of 11 prognosis-related and ADME-related genes. **C**, **D** The LASSO regression signature was constructed. **E** PCA of SARC samples based on the median risk score

1139 prognosis-related mRNAs were identified by ''survival'' package (Additional file 3: Table S3) [32]. Prognosis-related genes and ADME-related genes were intersected by R package ''veen'' and obtained 11 PARGs (Fig. 1A). The 11 PARGs were performed uni-Cox regression analysis (Fig. 1B). 265 patients with SARC were assigned to cohort train (*n* = 156) or test (*n* = 103), respectively. After selecting 7 genes by LASSO Cox regression analysis, a prognosis signature was constructed by the multi-Cox regression analysis (Fig. 1C, D). The risk score for each patient was calculated as follows:

$$risk \,score= -0.213902201617826*ALDH1A1-0.69129999454221*DHRS12-0.231751509847224*HNMT+0.498180587995638*PPARD+0.256169589090775*SLC16A1-0.212174514978431*SLC7A7-0.165647938855446*SOD3$$

Patients were assigned to high-risk or low-risk groups based on the median value of the risk score for all patients. Principal component analysis (PCA) showed that patients were distributed in two directions (Fig. 1E).

### Evaluation of prognostic signature

**Fig. 2:**
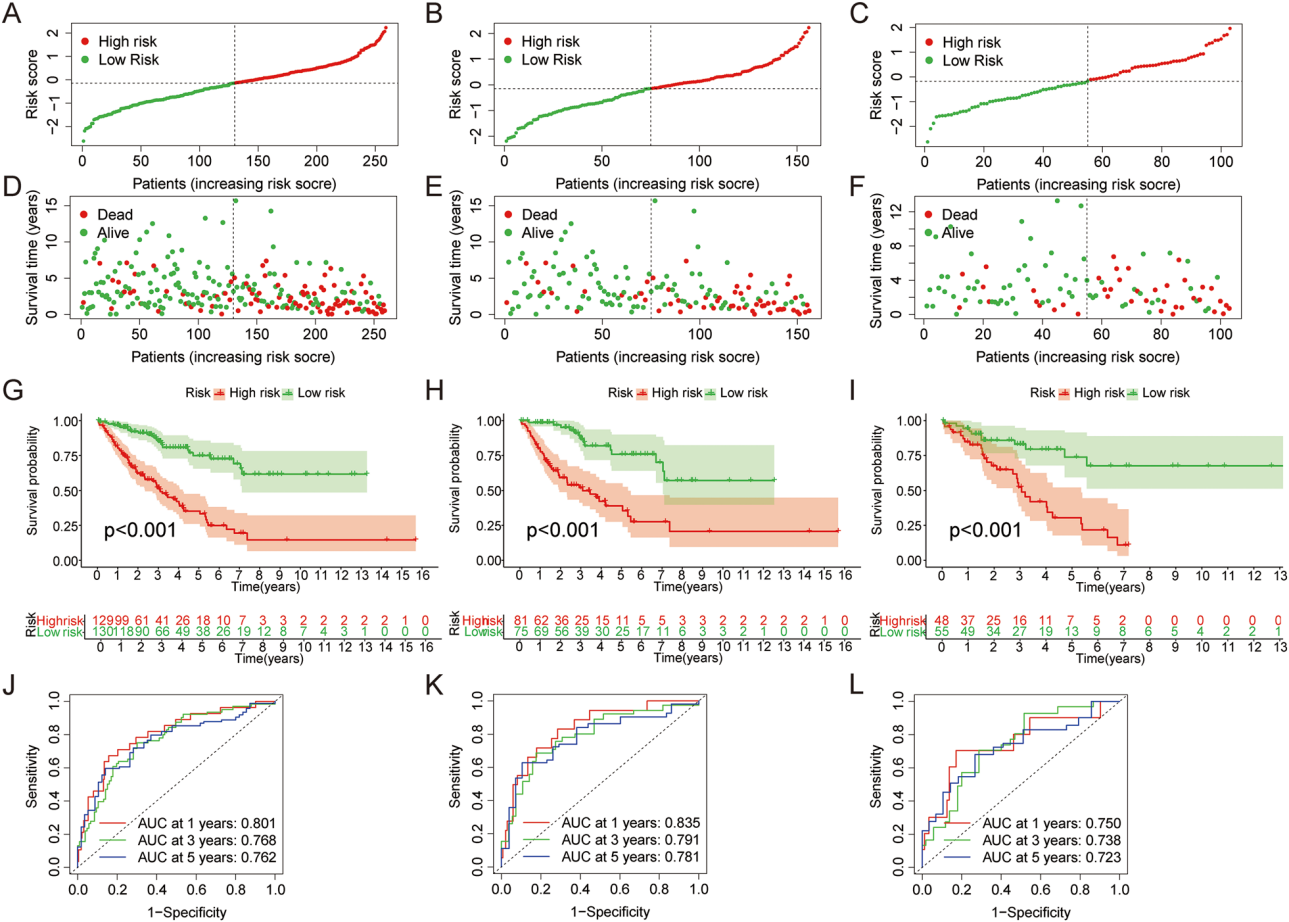
The prognostic ability of the signature. **A-F** Mortality in the entire, train and test cohorts, respectively. **G-I** Kaplan–Meier survival analysis of overall survival of SARC by in the entire, train and cohorts, respectively. **J-L** The ROC curve analysis was performed in entire, train and cohorts, respectively

The samples of entire TCGA cohort, train cohort and test cohort were divided into high-risk and low-risk groups based on median risk scores. Risk plots showed that patients with high-risk had shorter survival times compared to low-risk group (Fig. 2A-F). K–M survival curves showed that patients in the high-risk group had lower OS (Fig. 2G-I). Subsequently, ROC curves were performed to assess the predictive efficacy of the signature on survival. The area under curve (AUC) values for risk score for the entire cohort were 0.801, 0.768 and 0.762 at 1, 3 and 5 years, respectively (Fig. 2J). The AUC values for risk score for the train cohort were 0.835, 0.791, and 0.781 at 1, 3, and 5 years, respectively (Fig. 2K). The AUC values for risk score for the test cohort were 0.750, 0.738, and 0.723 at 1, 3, and 5 years, respectively (Fig. 2L).

### Evaluating prognostic signature in different subtypes of sarcoma

**Fig. 3:**
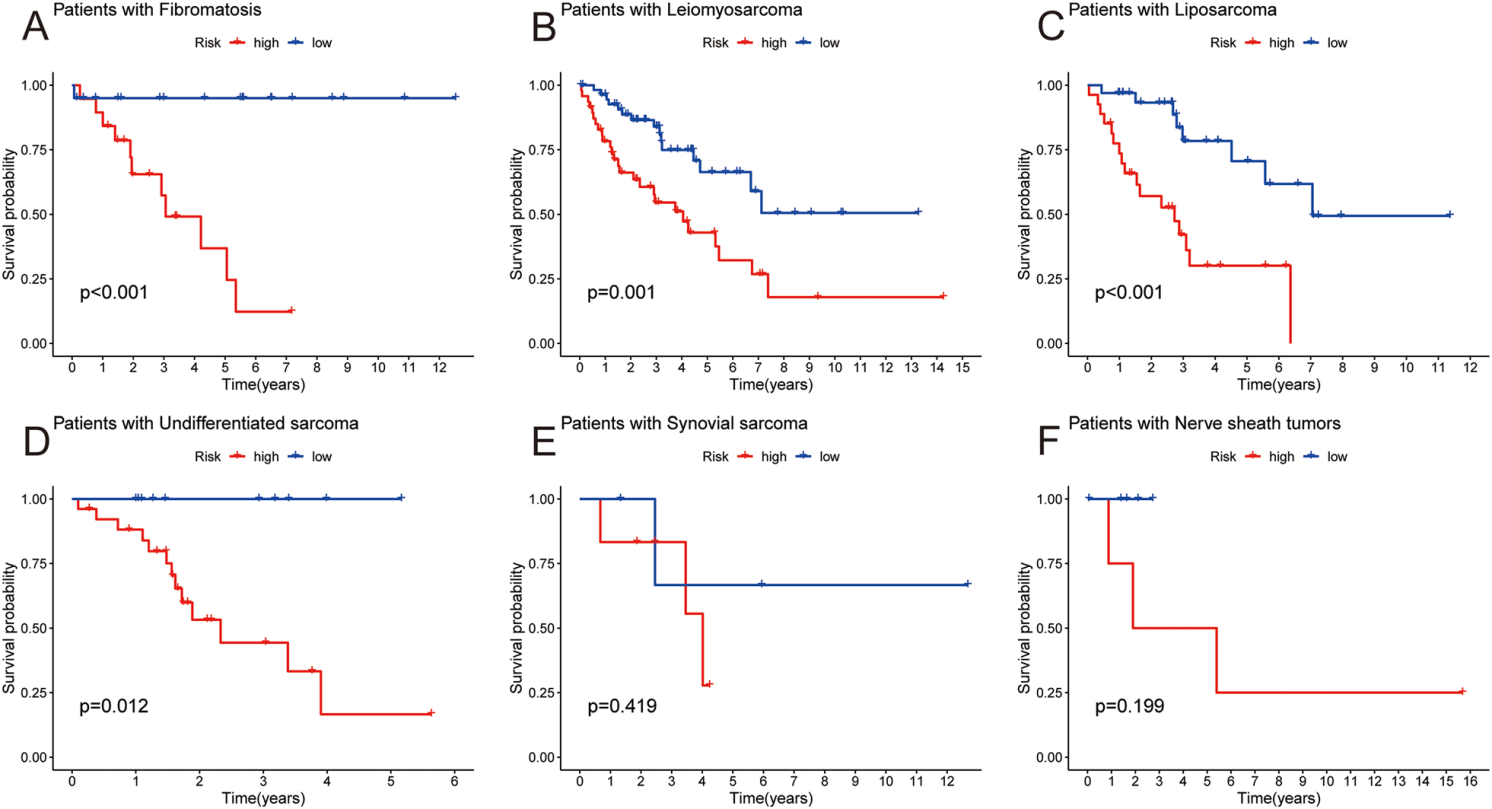
The prognostic value of prognostic signature in different subtypes of sarcoma. Kaplan–Meier survival analysis in different subtypes of sarcoma, including fibromatosis (**A**), leiomyosarcoma (**B**), liposarcoma (**C**), undifferentiated sarcoma (**D**), synovial sarcoma (**E**) and nerve sheath tumors (**F**)

Considering the heterogeneity of liposarcoma, the prognostic values of the signature in different subtypes of sarcoma were evaluated. The research results indicate that apart from synovial sarcoma and nerve sheath tumors, the signature has a good predictive ability for the remaining subtypes of sarcoma, including fibromatosis, leiomyosarcoma, liposarcoma, and undifferentiated sarcoma (Fig. 3).

### Predictive signature validation using TARGET-OS cohort

**Fig. 4:**
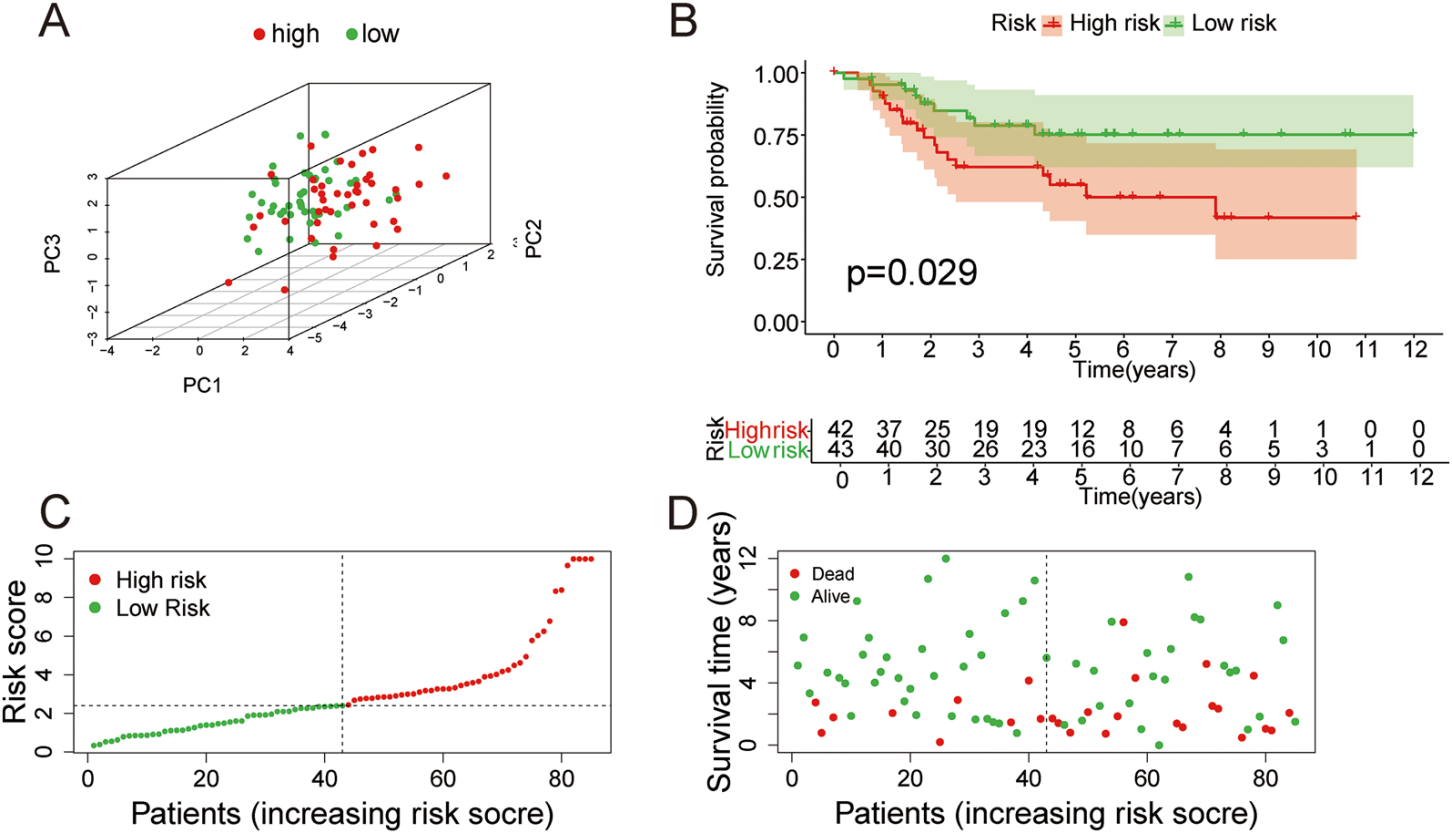
Validation of prognostic ability of the signature. **A** PCA of TARGET-OS samples based on the median risk score. **B** Kaplan–Meier survival analysis of overall survival of TARGET-OS cohort. **C**, **D** Mortality in the two risk groups in the TARGET-OS cohort

The TARGET-OS samples were treated as a validation dataset to test the reliability of the signature. PCA showed that patients were divided into two different directions (Fig. 4A). The high-risk patients had a poorer prognosis (Fig. 4B). Patients with high-risk scores had higher mortality (Fig. 4C, D).

### PPARD as a Carcinogen of Osteosarcoma

**Fig. 5:**
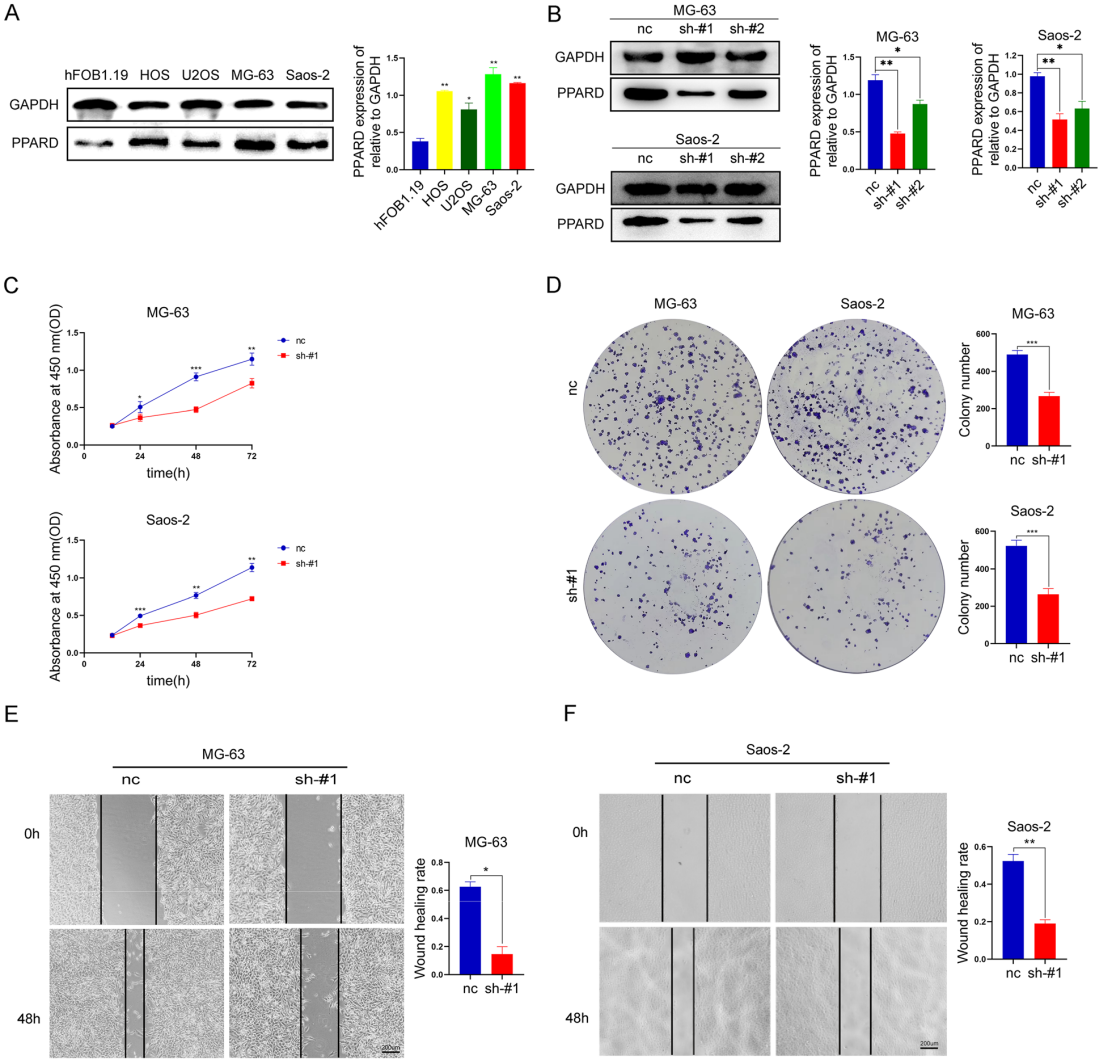
PPARD is raised in osteosarcoma and inhibits osteosarcoma cell proliferation and transfer. **A** Cellular lysates from osteoblasts and different osteosarcoma cell lines were collected for immunoblotting. The PPARD expression is quantified by ImageJ software in the corresponding column at right. **B** The expression level of PPARD in MG-63 and Saos-2 cells after knockdown. **C** Detection of proliferation rate of MG-63 and Saos-2 cells by CCK-8 after PPARD knockdown. **D** The ability of colony formation in MG-63 and Saos-2 cells after PPARD knockdown. Cell numbers are quantified in the corresponding column at right. **E**, **F** The wound healing showing the migration of MG-63 and Saos-2 cells after knockdown PPARD, scale bar = 200 μm. The results are quantified in the corresponding column at right

Because PPARD has the highest risk coefficient in uni-Cox analysis, it was selected for experimental verification in osteosarcoma. We first evaluated the protein expression profiles of PPARD in osteosarcoma cell lines and osteoblast. We found that PPARD was highly expressed in osteosarcoma cell lines, and the highest expression was found in MG-63 and Saos-2 cells (Fig. 5A). Next, we established PPARD knockdown stable cell lines in MG-63 and Saos-2, respectively (Fig. 5B). We found that sh-#1 knockdown effect was the most obvious, so we chose sh-#1 for subsequent experiments. Through CCK8, and cloning experiments, our data showed that the knockdown of PPARD significantly inhibited the proliferation of MG-63 and Saos-2 (Fig. 5C, D). The wound-healing assays presented inhibition of metastatic abilities of MG-63 and Saos-2 cells by PPARD knockdown (Fig. 5E, F).

### The status of immune cells infiltration in different risk groups

**Fig. 6:**
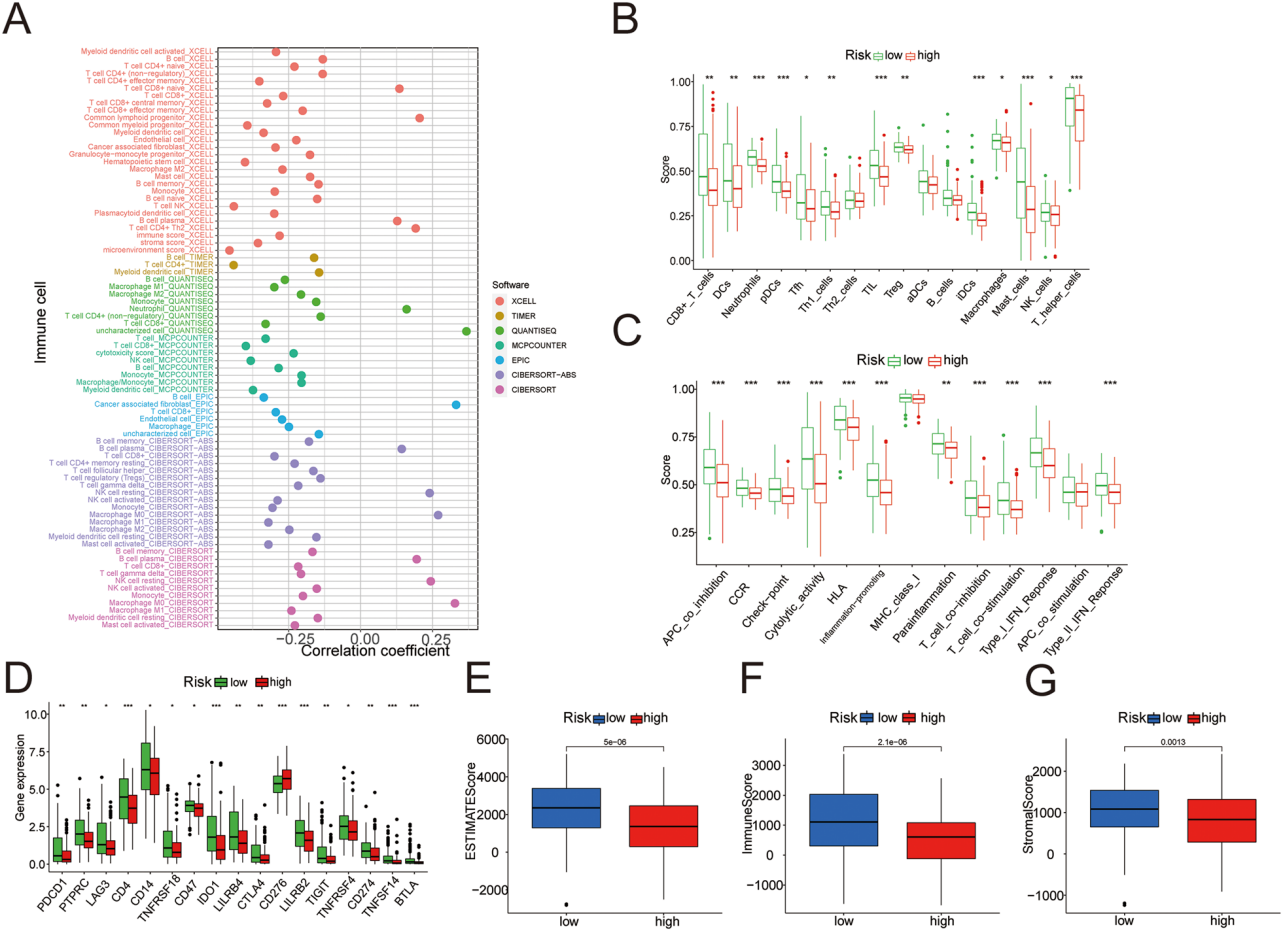
Differences in immune status between the two risk groups. **A** The bubble chart of correlation between immune cells and risk score. **B**, **C** The immune cell subtypes and immune-related functions and pathways were analyzed by single-sample gene set enrichment analysis. **D** The expression of immune checkpoints in the two risk groups. **E**–**G** The difference of immune microenvironment (ESTMATE, immune and stromal score) between high-risk and low-risk groups

To elucidate the relationship between risk score and the status of immune cell infiltration, bioinformatics analysis was performed with different algorithms by TIMER2.0 online tool. The results showed that different platforms and different immune cells were correlated with risk score differently, but they could roughly show a negative correlation between risk score and the status of immune cell infiltration (Fig. 6A). In other words, immunosuppression was present in the high-risk group, and the low-risk group was in a state of immune activation. In addition, the results of ssGSEA showed that the scores of immune cells and immune-related pathway were higher in the low-risk group (Fig. 6B, C). The differential expression analysis of immune checkpoint-related genes was performed and results showed that the expression of 17 genes (including CD-274 (PD-L1), PDCD-1 (PD-1) and CTLA4) was elevated in the low-risk group (Fig. 6D). Subsequently, we performed immune score (ESTIMATE score, immune score and stromal score) analysis, which showed higher results in the low-risk group (Fig. 6E-G).

### Immunotherapy and chemotherapy response

**Fig. 7:**
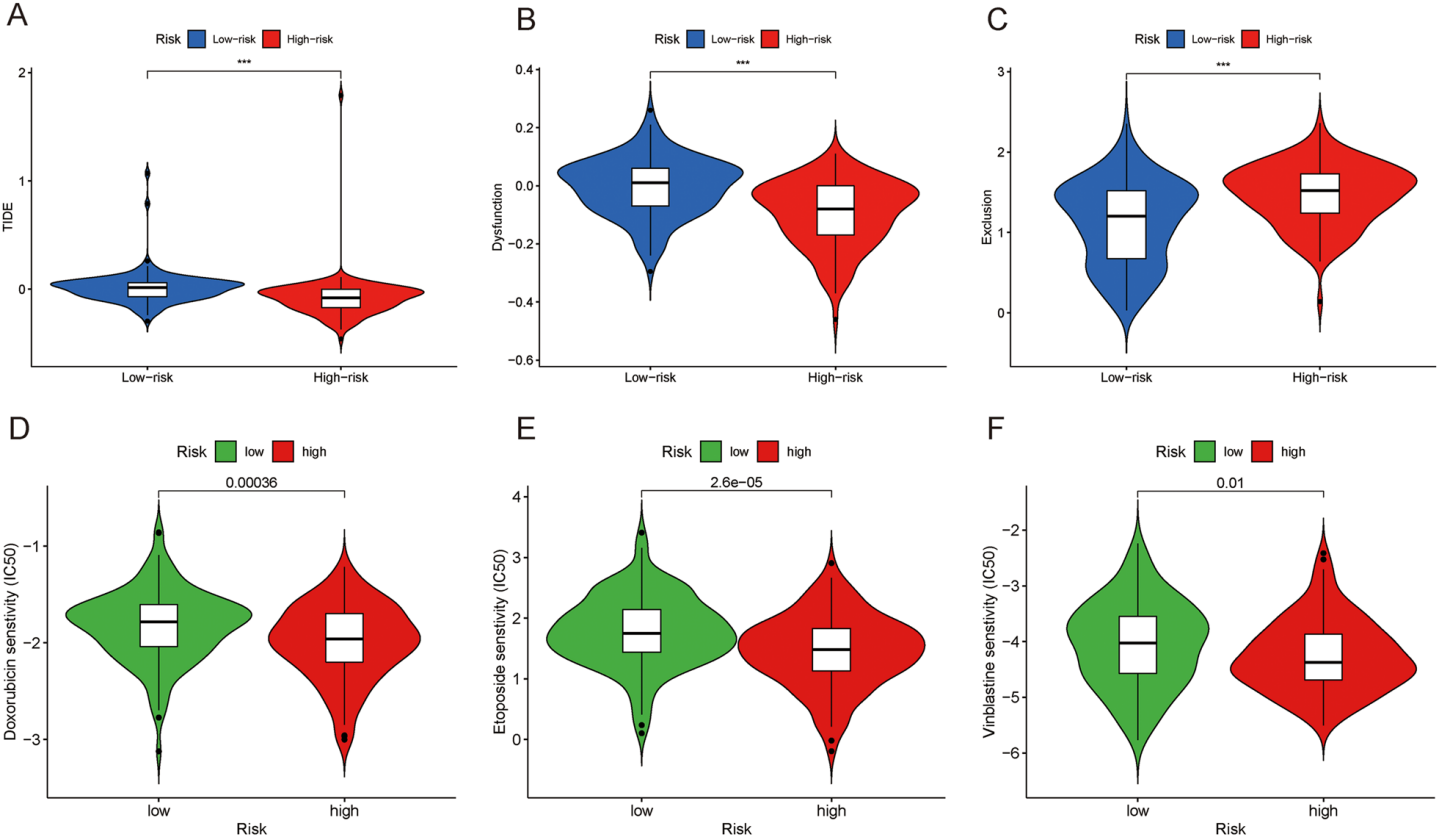
Prediction of immunotherapy. **A**-**C** The difference of TIDE score, T cell dysfunction score and T cell exclusion between the two risk groups. **D**-**F** The difference of sensitivity of three common chemotherapeutic drugs to the two risk groups

In this study, we evaluated the response of SARC patients to immune checkpoint blockade. The results showed that SARC with low-risk score had higher TIDE score and T cell dysfunction score than the patients with high-risk score (Fig. 7A, B). In contrast, the T cell exclusion score was higher in the high-risk group (Fig. 7C). This revealed that SARC patients with higher risk score had a better response to anti-immune checkpoint therapy. Subsequently, "pRRophetic" algorithm was performed to predict the sensitivity to chemotherapeutic drugs. Compared with low-risk group, high-risk group is more sensitive to doxorubicin, etoposide and vinblastine (Fig. 7D-F). In brief, we identified a PARGs-related signature to predict immunotherapy and chemotherapy response in SARC.

### Molecular subtypes constructed based on PARGs

**Fig. 8:**
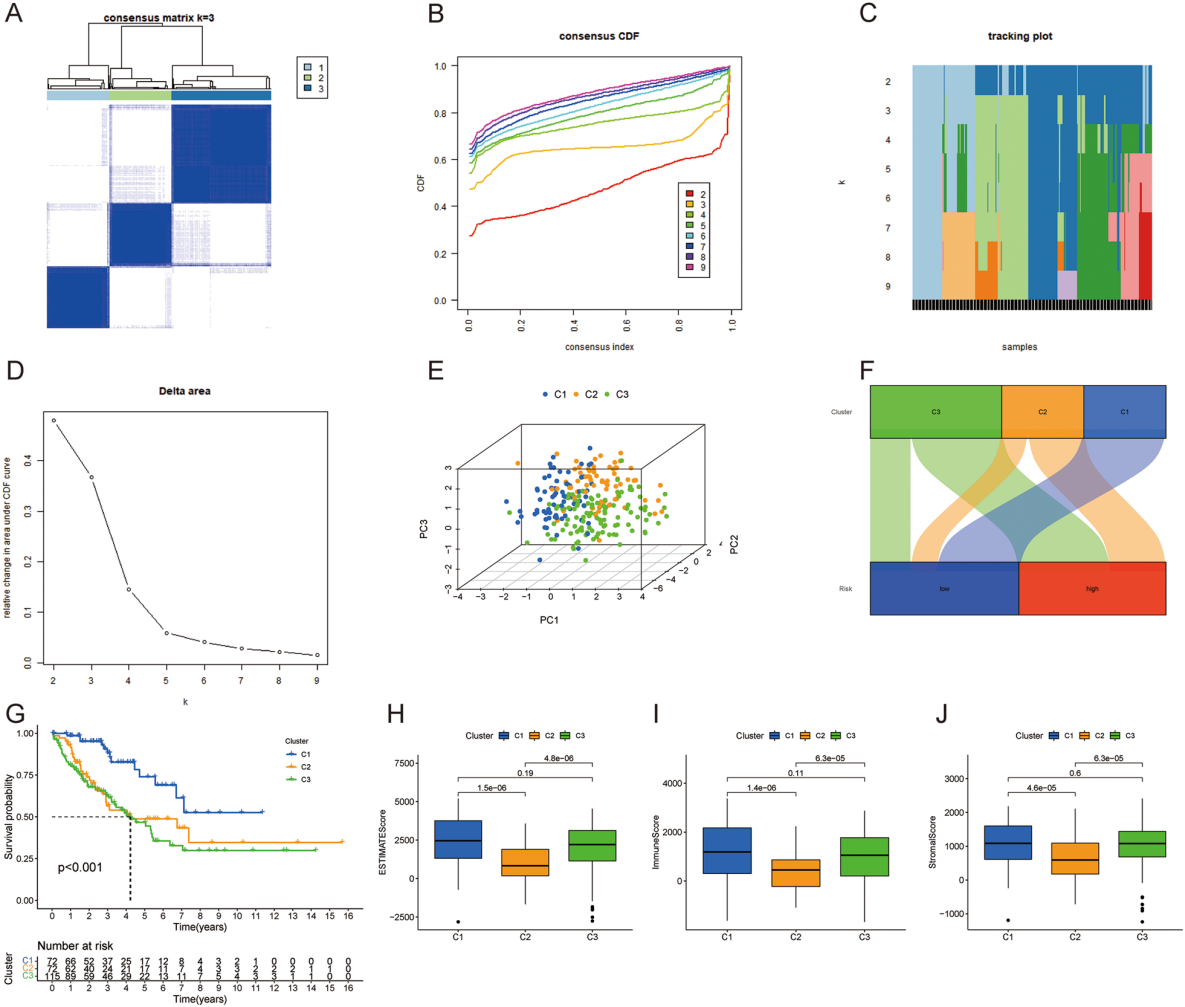
Consensus clustering of 7 PARGs. **A** Consensus clustering matrix for k = 3. **B** Consensus clustering CDF with k = 2–9. **C** The tracking plot for different k. **D** The area under the CDF curve for different k. **E** PCA of TARGET-OS samples according to the clustering. (F) The Sankey diagram of the two risk groups and the three clusters. **G** The survival estimates of the two clusters. **H**-**J** The difference of immune microenvironment (ESTMATE, immune and stromal score) in the three clusters

Consensus clustering was performed based on the expression profiles of the 7 PARGs. *k* = 3 was determined as the best cluster stability from *k* = 2 to 9, which showed maximum intra-group correlation and low inter-group correlation, indicating the feasibility of classifying patients into C1 (*n* = 72), C2 (*n* = 72) and C3 (*n* = 115) subtypes (Fig. 8A). Consensus cumulative distribution function (CDF) plots showed that the CDF reached an approximate maximum at *k* = 3 and that the classification was stable (Fig. 8B-D). PCA showed that the three clusters were clearly separated (Fig. 8E). Sankey plots showed that the C1 group corresponded to the low-risk group (Fig. 8F). Patients with Cluster1 had a better overall survival as shown by the K–M curve (Fig. 8G). By comparing the differences of TME score among the three cluster groups, we found that patients in the Cluster 1 group had higher TME scores (Fig. 8H-J).

## Discussion

Sarcoma is the largest and most heterogeneous group of rare cancers [33, 34]. The main pillar of radical SARC treatment is surgery, which is usually sufficient for low-grade tumors [35]. However, once metastasis occurs, the median survival of patients is often unsatisfactory [36]. Reliable and sensitive biomarker is essential for early diagnosis, inhibition of disease progression and improved prognosis.

In this study, we built a PARGs (ALDH1A1, DHRS12, HNMT, PPARD, SLC16A1, SLC7A7 and SOD3) signature through the TCGA-SARC cohort, and its ability to predict prognosis was verified in external TARGET-OS cohort. Patients in the high-risk group had shorter OS in both the TCGA-SARC and TARGET-OS cohorts. The results indicated that the PARGs signature is a valuable predictor of prognosis for SARC patients.

According to previous studies, these PARGs are involved in tumorigenesis. ALDH1A1, as a key ALDH1 isoenzyme associated with stem cell populations, is a cancer stem cell (CSC) marker and involves in self-renewal and differentiation in many solid tumors [37]. DHRS12 inhibits osteosarcoma proliferation and metastasis via the Wnt3a/β-catenin pathway [38]. SOD3 overexpression inhibits sarcoma cell proliferation, migration and metastasis [39].

PPARD is one of the three main isotypes of peroxisome proliferator-activated receptors (PPARs), which has been widely studied in cancer. The high expression of PPARD is associated with the activation of various oncogenic signaling pathways, including K-Ras, Wnt, and Src [40–42]. PPARD is up-regulated in many cancers, including lung cancer, colon cancer, and breast cancer [43–45]. Fatty acids, as natural active ligands of PPARD, could promote colorectal tumorigenesis [46]. PPARD promotes vascular repair after ischemia through its interaction with HIF1α [47]. PPARD agonists promote the expression of platelet-derived growth factor receptor beta, platelet-derived growth factor subunit B, and the tyrosinkinase KIT to promte tumor angiogenesis and progression [48]. We speculate that these are the reasons why it promotes osteosarcoma cell metastasis. Although some PARGs had been studied in SARC, their biological functions and molecular mechanisms remained unclear.

The sarcoma immune status can have a better understanding by biomarkers, which may help to decipher the reasons for different responses to immunotherapy [49–51]. Subsequently, immune cell infiltration in the tumor microenvironment was examined between the low-risk and high-risk groups. The results showed that the two groups had significantly different immune cell infiltration characteristics. Interestingly, we found that the level of immune cell infiltration, especially B and T cells, was higher in the low-risk group than in the high-risk group. The level of CD4 T cell infiltration was positively associated with the prognosis of patients with dedifferentiated liposarcoma [52]. These findings suggest that the PARGs signature can estimate the immune status of SARC patients. The higher level of infiltration of immune activated cells contributes to a better immune response against cancer cells, which could explain the improved prognosis observed in the low-risk group. Further assessing the relationship between the risk score and the benefits of immune checkpoint blockade therapy and chemotherapy, we found that patients with high-risk responded better to immune checkpoint blockade therapy compared to patients with low-risk. The high-risk group was more sensitive to the most commonly used chemotherapy regimens in SARC (doxorubicin, etoposide and vinblastine) and anti-immune checkpoint therapy.

In summary, a PARGs signature for SARC was constructed by the TCGA cohort and validated with TARGET-OS cohort. The risk signature performed well in predicting patient survival. In addition, we identified the relationship between PARGs-related risk score and immune cell infiltration in TME. The following analysis confirms the prediction of PARGs scores for response to immune checkpoint blockade therapy and chemotherapy. Our study provides a promising prognostic feature to guide the individualized treatment of SARC patients.

### Supplementary Information

**Additional file 1: Table S1.** The genes related to ADME.

**Additional file 2: Table S2.** The genes related to immune checkpoint blockers.

**Additional file 3: Table S3.** The mRNAs related to prognosis in sarcomas.

## Data Availability

The datasets presented in this study can be found in online repositories. The names of the repositories can be found in the article.
